# Biallelic and monoallelic pathogenic variants in CYP24A1 and SLC34A1 genes cause idiopathic infantile hypercalcemia

**DOI:** 10.1186/s13023-024-03135-8

**Published:** 2024-03-19

**Authors:** Qiao Wang, Jia-jia Chen, Li-ya Wei, Yuan Ding, Min Liu, Wen-jing Li, Chang Su, Chun-xiu Gong

**Affiliations:** Department of Endocrinology, Genetics and Metabolism, Beijing Children’s Hospital, 56# Nan Lishi Road, west district, Beijing, 100045 China

**Keywords:** Hypercalcemia, Nephrolithiasis, Idiopathic infantile hypercalcemia, CYP24A1, SLC34A1, Vitamin D, NaPi-II

## Abstract

**Objective:**

Idiopathic infantile hypercalcemia (IIH) is a rare disorder of PTH-independent hypercalcemia. *CYP24A1* and *SLC34A1* gene mutations cause two forms of hereditary IIH. In this study, the clinical manifestations and molecular aspects of six new Chinese patients were investigated.

**Methods:**

The clinical manifestations and laboratory study of six patients with idiopathic infantile hypercalcemia were analyzed retrospectively.

**Results:**

Five of the patients were diagnosed with hypercalcemia, hypercalciuria, and bilateral medullary nephrocalcinosis. Their clinical symptoms and biochemical abnormalities improved after treatment. One patient presented at age 11 years old with arterial hypertension, hypercalciuria and nephrocalcinosis, but normal serum calcium. Gene analysis showed that two patients had compound heterozygous mutations of *CYP24A1*, one patient had a monoallelic *CYP24A1* variant, and three patients had a monoallelic *SLC34A1* variant. Four novel *CYP24A1* variants (c.116G > C, c.287T > A, c.476G > A and c.1349T > C) and three novel *SLC34A1* variants (c.1322 A > G, c.1697_1698insT and c.1726T > C) were found in these patients.

**Conclusions:**

A monoallelic variant of *CYP24A1* or *SLC34A1* gene contributes to symptomatic hypercalcemia, hypercalciuria and nephrocalcinosis. Manifestations of IIH vary with onset age. Hypercalcemia may not necessarily present after infancy and IIH should be considered in patients with nephrolithiasis either in older children or adults.

## Backgrounds

Hypercalcemia is a relatively common clinical problem. Excluding primary hyperparathyroidism, tumor, vitamin D intoxication and low alkaline phosphatase, unexplained hypercalcemia, combined with the increase of urinary calcium excretion and suppressed parathyroid hormone (PTH), were previously named idiopathic infantile hypercalcemia (IIH). With the development of gene detection technology, the loss of function genetic variants in cytochrome P450 family 24 subfamily A member 1 (CYP24A1) gene and solute carrier family 34 member 1 (SLC34A1) gene have been identified as the molecular basis of IIH [1, 2].

*CYP24A1* genetic variants cause IIH type 1 (OMIM 143,880), which was first reported in 2011 [1]. *CYP24A1* gene encodes cytochrome P450 Family 24 Subfamily A Member 1. This mitochondrial protein initiates the degradation of 25-hydroxyvitamin D (25(OH)D_3_) and 1,25-dihydroxyvitamin D (1,25(OH)_2_D_3_) by hydroxylation of the side chain. Loss of CYP24A1 function blocks catabolism of 25-hydroxyvitamin D (25(OH)D_3_) and 1,25-dihydroxyvitamin D (1,25(OH)_2_D_3_). Accumulation of these active forms of vitamin D3 enhances intestinal Ca absorption and bone reabsorption, resulting in hypercalcemia, hypercalciuria, nephrocalcinosis, and suppressed intact parathyroid hormone. In 2016, Schlingmann et al. [2] described another type of IIH (IIH type 2), which was caused by loss of function mutation of *SLC34A1* and characterized by hypercalcemia, hypercalciuria, suppressed intact parathyroid hormone and hypophosphatemia. *SLC34A1* gene encodes a member of the NaPi-II family (NaPi-IIa), which plays a central role in phosphate reabsorption in the proximal tubule by using the sodium-electrochemical gradient to drive phosphate translocation against its concentration gradient [3]. *SLC34A1* mutations cause NaPi-II loss of function, and result in renal malabsorption of phosphorus and suppressed FGF23. Hypophosphatemia together with decreased concentration of FGF23 leads to the stimulation of 1α-hydroxylase (CYP27B1) and inhibition of CYP24A1, that results in an increment of 1,25(OH)_2_D_3_ levels, leading to hypercalcemia and hypercalciuria.

Both types of IIH present hypercalcemia, suppressed intact parathyroid hormone, hypercalciuria, and nephrocalcinosis. As nephrolithiasis is a common manifestation of IIH, IIH has been considered as a rare genetic cause of nephrolithiasis typically occurring in pediatric subjects with an estimated incidence of 1:33,000 live births [4]. Without timely and effective diagnosis and treatment, chronic renal failure may develop [5]. Only four cases have been reported in the Chinese region [6, 7]. Here we report six new Chinese patients with IIH, and describe the detailed clinical analysis, laboratory data collected, and the outcome after treatment.

## Results

### Clinical description

This study was conducted in 6 IIH patients diagnosed between 1 month and 11 years of age at the Beijing Children’s hospital of China from 2016 to 2022 (Table 1). Five patients (patient 1,2,3,5 and 6) were admitted with a suspected diagnosis of feeding difficulty, vomiting, poor weight gain, drowsiness and polyuria in infancy (Table 1). They all experienced symptoms and progressive exacerbation in early infancy. Among them, patient 5 was mistakenly applied cholecalciferol cholesterol emulsion (Vitamin D3 300000IU) at his 8 months old, which lead to a hypercalcemic crisis.

**Table 1:** Clinical and biochemical features of the six patients

		Patient 1	Patient 2	Patient 3	Patient 4	Patient 5	Patient 6		
**Sex**		M	F	M	F	M	M		
**Age of onset**		3 mo	3 mo	5 days	2 mo	1 mo	1 mo		
**Age at diagnosis**		9 mo	6 mo	1 mo	11 yr	8 mo	1 yr		
**Symptoms**	Fever					**+**			
	Feeding difficulty	**+**	**+**	**+**	**+**	**+**	**+**		
	Vomiting	**+**	**+**	**+**	**+**	**+**			
	Poor weight gain	**+**	**+**	**+**	**+**	**+**	**+**		
	Drowsiness	**+**							
	Deep respiration	**+**							
	Polydipsia	**+**							
	Polyuria	**+**							
	Urinary tract infection			**+**					
	Constipation			**+**		**+**	**+**		
	Hypertension				**+**				
**Physical examination**									
	Growth retardation	**+**	**+**	**+**		**+**	**+**		
	Dehydration		**+**			**+**			
	Hypotonia		**+**			**+**			
**History of vitamin D application**		400-500U/d (from neonatal period to 7 mo)			Oral calcium in infancy	Vitamin D 500IU/d in neonatal period, vitamin D3 300000IU once orally at 8 mo	vitamin D 500IU/d from 1 mo to 1 yr		
**Family history of renal calcification**		-	Mother	Father	-	Father Grandmother Aunt	Father		
**Laboratory findings**									
	**At initial presentation**							Normal range	
Serum Ca (mmol/L)		3.79	4.19	4.31	2.35	3.36	2.88	2.1–2.8	
Serum Pi (mmol/L)		1.52	1.05	1.57	1.72	1.19	1.15	1.37–1.99	
Serum Mag (mmol/L)		0.74	0.85	0.86	0.91	0.58	0.81	0.8–1.2	
ALP (U/L)		260	124	178	436	154	329	143–406	
PTH (pg/ml)		1.00	1.00	0.01	31.58	2	ND	10–69	
25(OH)D_3_ (nmol/L)		30.8	25.3	24.4	ND	> 400	77.4	≥ 50	
1,25(OH)_2_D_3_ (pg/ml)		ND	ND	ND	110	ND	ND		
Urine Ca/Cr (mmol/mmol)		1.67	ND	ND	3.19	1.35	0.24	0.00-0.20	
24 h urine Ca (mmol/kg/24 h)		0.22	0.22–0.31	0.17–0.22	ND	0.21	ND	< 0.2	
GFR (mL/min/1.73 m2)		84.2–127.0	42.5–94.7	45.9	89.7	66.9-136.3	104.2	< 90	
	**At last observation**								
Age		2 year 6 mo	6 year 8 mo	4 mo	13 year	1 year 5 mo	1 year 5 mo		
Serum Ca (mmol/L)		2.2	2.40	2.55	2.45	2.56	2.45	2.1–2.8	
Serum Pi (mmol/L)		ND	1.57	1.9	ND	1.87	1.37	1.37–1.99	
ALP (U/L)		ND	270	320	ND	ND	44.8	143–406	
PTH (pg/ml)		ND	ND	1.2	ND	ND	ND	10–69	
Urine Ca/Cr (mmol/mmol)		ND	0.09	0.44	0.16	ND	0.09	0.00-0.20	
GFR (mL/min/1.73 m2)		ND	131.7	100.6	77.7	ND	137.1	< 90	
**Imaging examination**									
Nephrocalcinosis		**+**	**+**	**+**	**+**	**+**	**+**		
Skeletal abnormality		**-**	**-**	**+**	**-**	**+**	ND		
Parathyroid ultrasound		**-**	**-**	**-**	ND	**-**	ND		
**Gene analysis**		*CYP24A1* c.116G > C het	*SLC34A1* c.1322 A > G het	*SLC34A1* c.1697_1698insT het	*CYP24A1* c.1349T > C and c.287T > A	*CYP24A1* c.823T > C and c.476G > C	*SLC34A1* c.1726T > C het and *CYP24A1* c.376 C > T het		

*Ca: calcium; Pi: phosphorus; Mag: magnesium; ALP: alkaline phosphatase; ALB: albumin; GFR: Glomerular filtration rate; ND: not done

One girl (Patient 4) was diagnosed at 11 years with arterial hypertension and nephrocalcinosis. She presented feeding difficulty, vomiting, and slow weight gain at 2 months old, but she didn’t test blood or urine electrolytes at that time. The symptoms improved spontaneously at 10 months old. When she was 6 years old, bilateral renal medullary calcification was determined by ultrasound. When she was 11 years old, she was referred for elevated blood pressure (130/80mmHg) and her creatinine clearance rate was at a lower limit (89.7 ml/(min*1.73m^2^).

**Fig. 1:**
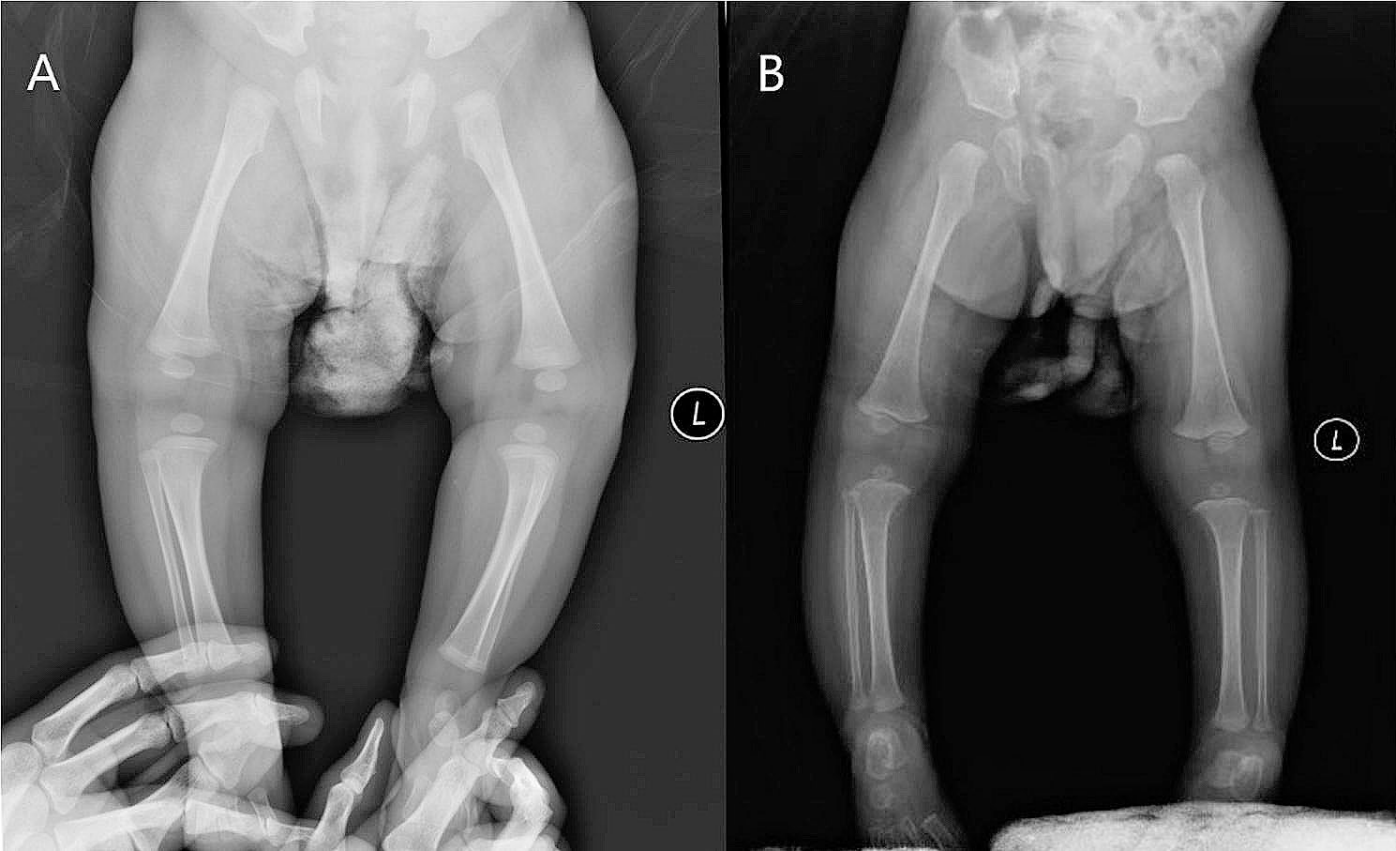
X-ray examination of limbs. (**A**) Long bone panorama of Patient 3 showed dense transverse banded shadows in Bilateral distal femur and tibial metaphysis. (**B**) Radiography of Patient 5 showed temporary calcification zone is slightly narrow and dense of in the metaphysis of the bone

The clinical characteristics of the patients are displayed in Table 1, and Fig. 1 showed bone X-ray of the patients.

### Treatment and follow-up

**Fig. 2:**
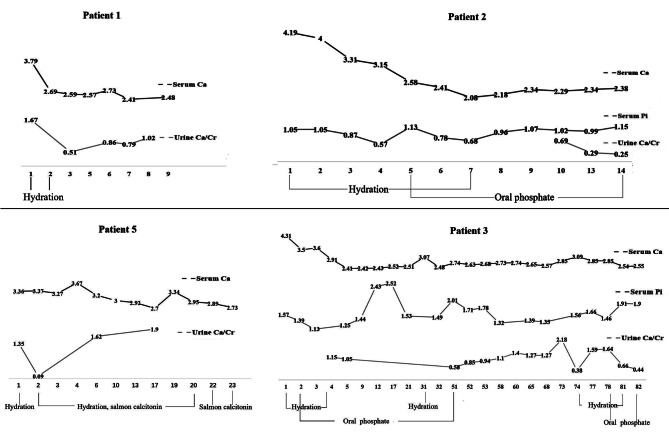
Changes of serum calcium, phosphorus and urine calcium during hospital. Line charts showed levels of serum calcium, rates of urinary calcium excretion and serum phosphorus during hospital. The numbers in the line charts represents the measured values, and the unit of calcium and phosphorus is mmol/L, and the unit of urine Ca/Cr is mmol/mmol

All patients stopped taking vitamin D and calcium preparations and avoided sunlight after discharge. The symptoms of patients 1, 2, 3 and 6 improved within a few days with hydration treatment, and the levels of serum calcium gradually decreased to normal (Fig. 2). Judging from the curves of serum calcium and urine calcium, the decrease of serum calcium levels is earlier than urine calcium, and patients still have persistent hypercalciuria when their blood calcium dropped to normal (Fig. 2). Hypercalcemic crisis in Patient 5 is difficult to be treated. His blood calcium fluctuates after hydration, furosemide, calcitonin and hemodialysis treatments. Finally, three months after diagnosis, his blood calcium decreased to normal after bisphosphonate therapy.

Among the 6 patients, patient 3 was lost to follow up, and the follow-up time for the other patients was 5 months to 2 years. During follow-up, Patient 1, 2, 5 and 6 remained asymptomatic with normal serum calcium level during follow-up, their physical and intellectual development were normal. Serum calcium level of Patient 4 remained normal during monitoring. However, her blood creatinine level is higher two years later, creatinine clearance rate declined to 77.7 ml/(min*1.73m^2^). Her blood pressure fluctuated within the range of 120–130/75-80mmHg.

### Gene results

**Fig. 3:**
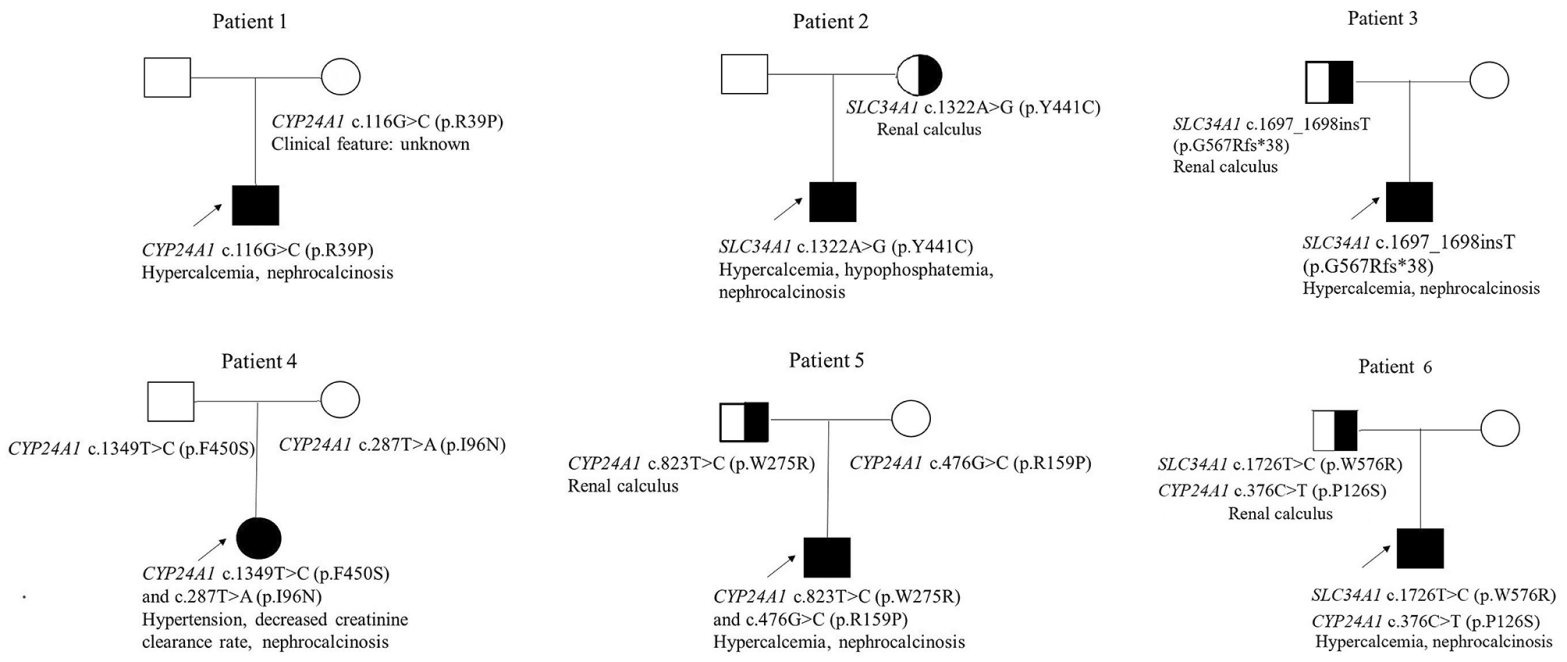
Pedigree of the patient’s family. The affected patients are shown as a filled square

All patients underwent next-generation sequencing. Mutations in *CYP24A1* or *SLC34A1* gene were identified in all patients, combined with clinical manifestations, conforming to the diagnosis of IIH. The genetic test results of the patients and the verification results of their parents are shown in Fig. 3. Patient 4 and Patient 5 were found have biallelic mutations of *CYP24A1*. Patient 1 was found have a heterozygous *CYP24A1* mutation (c.116G > C) and patient 2 and patient 3 each have a heterozygous *SLC34A1* mutation (c.1322 A > G and c.1697_1698insT, respectively). Patient 6 simultaneously have a mutation of *CYP24A1* and a mutation of *SLC34A1*, and both mutations originated from his father. Parents’ carriers of patient 2,3 and 6 have renal calcification.

Six *CYP24A1* variants (c.116G > C, c.287T > A, c.376 C > T, c.476G > A, c.823T > C, and c.1349T > C) and three *SLC34A1* variants (c.1322 A > G, c.1697_1698insT and c.1726T > C) were found. *CYP24A1* c.823T > C (p.W275R) and c.376 C > T (p.P126S) were previously reported in patients with hypercalcemia [8]. The other four *CYP24A1* mutations and three *SLC34A1* mutations have not been reported before.

According to Standards and guidelines for the interpretation of sequence variants: a joint consensus recommendation of the American College of Medical Genetics and Genomics and the Association for Molecular Pathology, *CYP24A1* c.1349T > C (p.F450S), c.476G > C (p.R159P) and *SLC34A1* c.1322 A > G (p.Y441C), c.1726T > C (p.W576R) are of uncertain significance (PM2 + PP3), with extremely low frequency and were predicted as “damaging” to the protein by SIFT, PolyPhen_2, MutationTaster, GERP + + and REVEL. *CYP24A1* c.287T > A (p.I96N) was reported with MAF (Minimum allele frequency) of 0.0017 (0.17%) in East Asians, and is predicted as “damaging” with software evidence of conservation and protein structure prediction (PP3). *CYP24A1* c.116G > C (p.R39P) was predicted as PM2 + BP4. *SLC34A1* c.1697_1698insT (p.G567Rfs*38) is located within exon 13, which causes a gene frameshift and results in a premature termination codon.

## Discussion

**Fig. 4:**
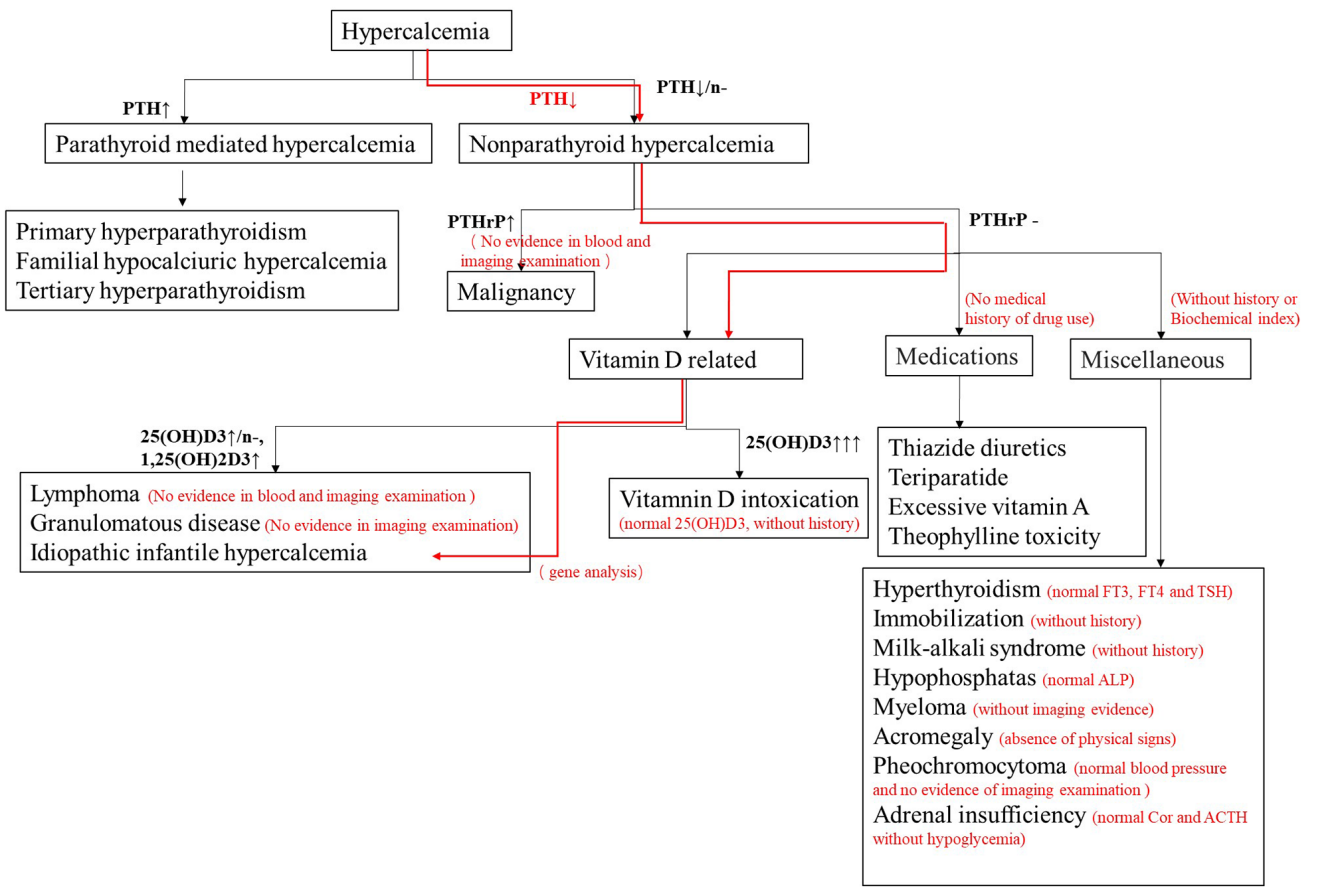
Diagnosis flow chart of hypercalcemia. The black line represents the diagnosis process of hypercalcemia in general, and the red line represents the diagnosis process of patients in this study. As PTHrP cannot be tested in China at present, and some patients do not have record of 1,25(OH)_2_D_3_ level, assistance is given according to the medical history and related biochemical indicators and imaging examination to exclude other diseases

In this article we report six Chinese patients with IIH, that were subjected to molecular and genetic analysis, and their IIH diagnosis was additionally verified by clinical and biochemical manifestations (Fig. 4). Among the six patients, five presented typical infantile hypercalcemia, while one patient (Patient 4) did not show hypercalcemia at diagnosis, but hypercalciuria was still obvious when she was 11 years old with a low-normal creatinine clearance rate. These data reflect the different manifestations of IIH at different life stages. Hypercalcemia can be temporary. The prognosis of hypercalciuria varies. In previous report, the hypercalciuria of IIH was difficult to improve [9]. Our patient 4 still had hypercalciuria at the age of 11 years, however urinary calcium levels of our patient 2 gradually decrease to normal during follow-up. Besides the pathogenicity of gene mutations, IIH phenotype is also influenced by many environmental factors like diet, lifestyle, vitamin D intake, and activity of the other vitamin D metabolism enzymes [10]. Renal maturation may contribute to the onset of IIH, and haploinsufficiency is more obvious in infancy, suggesting that the occurrence of diseases caused by heterozygous variants is related to age [11]. However, nephrocalcinosis is a consistent manifestation of IIH, as was previously reported [9]. In patient 3, an ultrasound showed bilateral renal nephrocalcinosis at one-month-old, which occurred very early, probably before birth. In patient 4, nephrocalcinosis was first found at 6 years old and remained when she was 11 years old. There is insufficient evidence supporting that nephrocalcinosis tends into remission spontaneously with age. Renal calcification may be the only manifestation of the later stages of IIH [5]. The GFR of patient 4 was lower than 90 ml/min/1.73 m^2^. Janiec A et al. [5] showed that IIH patients have a greater risk of progressive chronic kidney disease, with a rate of 77%. The severity of the initial kidney injury rather than nephrocalcinosis appears to play a significant role as a trigger of progressive chronic kidney disease (CKD). Patient 4 presented feeding difficulty, vomiting and slow weight increase when she was 2 months old, however, GFR wasn’t recorded. The other patients’ GFR were low when admitted, and their GFR increased to normal range after hydration treatment. Renal function monitoring is needed in long-term follow-up.

In IIH patients, treatment included removing vitamin D supplementation, a low-Ca diet, and Pi supplementation in NaPi-IIa defect patients [12]. Patients with hypercalcemic episodes may be treated with hydration and diuretics. If symptomatic hypercalcemia persists, bisphosphonates, calcitonin, glucocorticoids, and hemodialysis can be administered [13]. In patients 1, 2, 3 and 6, hydration and diuretics reduced the blood calcium level to normal within a few days. Patient 5 still had persistent hypercalcemia, which could not be reduced to normal range after adding calcitonin or hemodialysis. Patient 5 began to receive routine vitamin D (500u/d) treatment after birth. He then gradually developed feeding difficulty and astriction, reflecting hypersensitivity of IIH patients to vitamin D supplementation. However, patient 5 was given oral administration of Cholecalciferol Cholesterol Emulsion (300,000 units of vitamin D3) at 8 months old without testing for vitamin D or serum calcium level. Following this treatment, his hypercalcemia symptoms aggravated and his 25(OH)D_3_ level exceeded 400nmol/L, meeting the diagnostic criteria of intoxication by the Endocrine Society [14]. Combined vitamin D intoxication aggravates treatment difficulties. We agree with the view that empirical therapy of vitamin D deficiency with high vitamin D doses is discouraged without previous documentation of 25(OH)D_3_ concentrations and monitoring of 25(OH)D_3_ and serum calcium levels [15]. In the absence of detection, a single high dose of vitamin D given to patients with IIH can cause serious consequences. Due to renal calcification being a common manifestation of IIH, urinary ultrasound is a useful tool to document nephrocalcinosis and should be done before implementing such treatment.

IIH type 1and type 2 are described as an autosomal recessive disorder, however, individuals of a single heterozygote presenting chronic and latent symptoms have also been reported [16]. In our patients, biallelic variants of *CYP24A1* were found in two patients, while four were found to have mono mutant allele of *CYP24A1* and/or *SLC34A1*. Functional validation tests are needed, however, clinical evidence helped confirm the disease.

In 2011, Schlingmann et al. [1] first reported biallelic mutations of *CYP24A1* were agenetic cause of IIH. Cases with monoallelic variants in *CYP24A1* gene were noticed and suggested an autosomal dominant inheritance with reduced penetrance [11, 17]. Here, our patient 1 with monoallelic *CYP24A1* variant presented symptomatic hypercalcemia in infancy, providing a clinical reference for the view of the potential risk of developing hypercalcemia and related clinical manifestations if exposed to triggering factors [18]. Unlike the cases of Molin A et al. [11] reported without renal disease, our patient 1 has significant renal calcification. In addition, the father of patient 5 carrying *CYP24A1* mutation had kidney calculus, as well as his mother and sister. However, we cannot obtain DNA samples from the two women to verify whether their kidney calculus is related to the mutation.In a family survey by Brancatella A et al. [18], the rate of nephrolithiasis showed no difference between heterozygotes and the wild-type subjects, however, serum total calcium concentrations and 25(OH)D_3_ concentrations were significantly higher in heterozygotes than in the wild-type subjects. These clinical cases reflect that monoallelic variants in *CYP24A1* gene can cause infantile hypercalcemia, but the presence of renal calcification is still controversial. More clinical and laboratory evidence needs to summarize and the molecular mechanism needs to be explored.

The phenotype of IIH induced by *SLC34A1* mutations (IIH type 2) was first described by Schlingmann et al. [2] in 2016, which is characterized by infancy onset with failure to thrive, polyuria, and medullary nephrocalcinosis. Hypercalcemia, suppressed PTH, hypophosphatemia, and impairment of renal phosphate conservation were demonstrated in laboratory data. IIH type 2 was also described as a recessive disease. Monoallelic heterozygous variants in *SLC34A1* were first described to cause hypophosphatemic nephrolithiasis/osteoporosis-1 (NPHLOP1), Prie, D et al. [19] reported one patient and her only daughter showed symptoms and proposed the dominant inheritance of the disease. Schlingmann et al. [2] also described some heterozygous relatives of IIH type 2 patients having nephrolithiasis. However, no more clinical evidence was reported. Our patients with monoallelic *SLC34A1* variant also presented with symptomatic hypercalcemia. Additionally, their parents carrying the *SLC34A1* mutation were also found having nephrocalcinosis. Our reports provided clinical evidence of the effect of monoallelic heterozygous variants in *SLC34A1.* Increased urinary phosphate and calcium excretion, elevated plasma 1,25(OH)_2_D_3_ and urolithiasis were shown in heterozygous *SLC34A1*-deficient mice (*SLC34A1*+/-), indicating the dominant negative effect of the mutant *SLC34A1* protein on the function of the wildtype [19]. The underlying mechanism of the dominant-negative effect needs further exploration. Patient 6 and his father were found carrying heterozygous c.1726T > C in *SLC34A1* gene and heterozygous c.376C > T in *CYP24A1* gene. Combined with the patient’s hypercalcemia and hypophosphatemia, heterozygous c.1726T > C in *SLC34A1* is considered responsible for IIH. Heterozygous c.376C > T in *CYP24A1* gene might contribute to the severe phenotype of both patients.

Although the reported data are not sufficient for a final evaluation of the genetic mode of *CYP24A1* and *SLC34A1*-related hypercalcemia, clinical evaluation and long-term observation are important for patients carrying monoallelic variants. Only four IIH patients have been reported in the Chinese population [6, 7], who were identified with compound heterozygous mutations, without case report of monoallelic variants. Gene reports tend to ignore heterozygous variations. The frequency of kidney stones due to CYP24A1 deficiency was estimated between 420 and 1960 per 10,000 [20]. According to *Expert Consensus on Clinical Application of Vitamin A and Vitamin D in Chinese Children*, vitamin D 400-800IU per day is routinely supplemented after birth [21]. If we disregarded *CYP24A1* variant carriers, a regular dose of vitamin D supplementation may promote the formation of renal medullary calcification. Investigation of *CYP24A1* and *SLC34A1* mutation frequency in the Chinese population and monitoring of blood calcium and urinary calcium during routine vitamin D supplementation need to be further explored.

Here, we described the clinical, biochemical, and genetic manifestations of six Chinese patients with IIH. Our study has certain limitations. Our study extended over a short period of time and included a limited sample size. Therefore, a cohort study with a long-term outcome of a larger sample size is needed in the future.

In conclusion, manifestations of IIH were different with age. Hypercalcemia and hypercalciuria can be gradually relieved, but nephrocalcinosis starts early and persists. Transient high calcium symptoms in infancy may go un-noticed in adult patients with nephrolithiasis. In addition, our reports provided some clinical evidence of the pathogenicity of monoallelic heterozygous variants in *CYP24A1* and *SLC34A1*, suggesting that the monoallelic heterozygous *SLC34A1* or *CYP24A1* variant also contributes to symptomatic IIH.

## Methods

All parents had signed an informed consent form for using patients’ data. This study was approved by the ethics committee of Beijing Children’s hospital, Capital Medical University, Beijing, China. Ethical approval ID: IEC-C-006-A04-V.06.

Next generation sequencing.

Genomic DNA was extracted from peripheral blood leucocytes using QIAamp DNA Blood Midi kit (Qiagen, Hilden, Germany). Sequences were generated using the Agilent Bioanalyzer. Next generation sequencing was performed on an Illumina HiSeq 2000 platform. After HiSeq 2000 sequencing, high-quality reads were retrieved from raw reads by filtering out the low quality reads and adaptor sequences using the Solexa QA package and the cutadapt program (http://code.google.com/p/cutadapt/), respectively. SOAP aligner program was- to align the clean read sequences to the human reference genome (hg19). Sanger sequencing validation was performed for all patients found to harbour a gene mutation and for their affected siblings. Forward and reverse primers were manually designed on the flanking mutated regions. PCR amplification was optimized in accordance to the standard PCR protocol using FastStart Taq DNA Polymerase, dNTPack (Roche Applied Science). Sequencing reaction was performed using the BigDye® v.1.1 Terminator cycle sequencing kit and the ABI Prism® 3130xl Genetic Analyzer (Life Technologies).

## Data Availability

The data that support the findings of this study are available on request from the corresponding author. The sequence data have been deposited in the Genome Sequence Archive (Genomics, Proteomics & Bioinformatics 2021) in National Genomics Data Center (Nucleic Acids Res 2022), China National Center for Bioinformation / Beijing Institute of Genomics, Chinese Academy of Sciences (https://ngdc.cncb.ac.cn/gsa-human/browse/HRA004188). Data reported in this paper will be shared by request to Data Access Committee via GSA-Human System.

## Abbreviations

CYP27B1: 1α-hydroxylase
1,25(OH)2D3: 1,25-dihydroxyvitamin D
CYP24A1: cytochrome P450 family 24 subfamily A member 1
NPHLOP1: hypophosphatemic nephrolithiasis/osteoporosis-1
IIH: idiopathic infantile hypercalcemia
NaPi-II: Na+-coupled Pi cotransporters
PTH: parathyroid hormone
SLC34A1: solute carrier family 34 member 1
